# Extrapulmonary Tuberculosis among Somalis in Minnesota^1^

**DOI:** 10.3201/eid1209.050295

**Published:** 2006-09

**Authors:** R. Bryan Rock, Wendy M. Sutherland, Cristina Baker, David N. Williams

**Affiliations:** * University of Minnesota Medical School, Minneapolis, Minnesota, USA;; † Hennepin County Medical Center, Minneapolis, Minnesota, USA;; ‡ Minnesota Department of Health, Saint Paul, Minnesota, USA

**Keywords:** Tuberculosis, immigrants, Minnesota, Somalia

## Abstract

To analyze extrapulmonary tuberculosis in Somalis living in Minnesota, we reviewed surveillance and public health case management data on tuberculosis cases in ethnic Somalis in Minnesota from 1993 through 2003. The presence of these recent immigrants substantially affects the local epidemiology and clinical manifestation of tuberculosis.

Although the incidence of tuberculosis in the United States has declined each year since 1993, tuberculosis remains an important infectious disease in the United States and worldwide. In Minnesota, the incidence of tuberculosis increased during the 1990s and peaked at 4.9 cases per 100,000 population in 2001. From 2001 through 2005, 81% of tuberculosis cases in Minnesota occurred in foreign-born persons; this finding can largely be attributed to dynamic immigration patterns that have included an influx of persons from areas of the world where tuberculosis is endemic (*1*).

Somalia ranks in the top 15 countries of origin for foreign-born persons with cases of tuberculosis in reported in the United States (*2*). Minnesota has the largest Somali population in the United States (*3*). Although Somali persons constitute <1% of Minnesota's population, they accounted for 30% of tuberculosis cases reported statewide from 1999 through 2003. The unique epidemiologic characteristics of foreign-born tuberculosis patients in Minnesota, and Somali tuberculosis patients in particular, have been described (*1*,*4*).

The emergence of extrapulmonary disease as an important form of active tuberculosis has been noted in many studies (*5*–*7*). Our purpose was to describe the characteristics of extrapulmonary tuberculosis in ethnic Somalis in Minnesota and to assess factors that may contribute to its disproportionately high prevalence in this population.

## The Study

Data were obtained from the Minnesota Department of Health's tuberculosis database, specifically, surveillance and public health case management data on all cases of tuberculosis reported among ethnic Somalis in Minnesota from January 1, 1993, through December 31, 2003. Cases were defined in accordance with the Centers for Disease Control and Prevention's surveillance case definition for tuberculosis (*8*).

Of the 407 cases of tuberculosis in ethnic Somalis reported to the Minnesota Department of Health during this 10-year period, 239 (59%) had extrapulmonary involvement, including 198 (49%) with exclusively extrapulmonary disease and 41 (10%) with pulmonary and extrapulmonary tuberculosis. The remaining 168 (41%) patients had pulmonary disease only.

In 2003, 214 cases of tuberculosis (4.4 cases per 100,000 population) were reported in Minnesota; 173 (81%) of these patients were foreign-born, and 58 (27%) were from Somalia. Of the 58 Somali patients, 45 (78%) had extrapulmonary disease. According to US Census data, an estimated 11,164 ethnic Somalis were residing in Minnesota in 2000 (*9*); by June 2004, this population had increased to ≈25,000 (*9*). Based on these numbers, the approximate annual incidence rate of tuberculosis for Somalis in Minnesota in 2003 was 269 cases per 100,000 population, and the approximate rate of extrapulmonary tuberculosis was 209 cases per 100,000 population.

Demographic and clinical characteristics of the 239 Somali patients who had extrapulmonary tuberculosis are summarized in the Table. A total of 179 (75%) patients were tested for HIV within 1 year of the diagnosis of tuberculosis; HIV test results were positive for only 2 (1%).

The only characteristics that differed significantly between patients with extrapulmonary tuberculosis and pulmonary tuberculosis were age and length of time in the United States before diagnosis. Patients who had extrapulmonary tuberculosis were generally older (mean 26.8 years) than those with pulmonary tuberculosis (mean 23.7 years) (p = 0.01). Similarly, the length of time between arrival in the United States and diagnosis of tuberculosis was generally longer for patients with extrapulmonary tuberculosis (mean 2.7 years) than for those with pulmonary disease (mean 1.3 years) (p<0.00001). In a logistic regression model that controlled for the confounding effects of length of time in the United States on the association between a patient's age and risk for extrapulmonary disease, only the patient's duration of residence in the United States was significantly associated with extrapulmonary tuberculosis (p<0.00001). Controlling for age, each additional year of residence in the United States was associated with a 30% increase in the risk for extrapulmonary tuberculosis (odds ratio 1.3, 95% confidence interval 1.2–1.5).

A total of 250 sites of extrapulmonary disease were identified in the 239 patients, representing 26 distinct anatomic locations of disease; several patients had disease in multiple sites: lymph nodes (50%), pleura (9%), peritoneum (8%), skin or soft tissue (8%), central nervous system (6%), bones or joints (4%), various organs (miliary) (4%), urogenital tract (2%), and other sites (9%).

Among the 197 (82%) cases of culture-confirmed extrapulmonary tuberculosis, 18% were resistant to >1 first-line antituberculosis drug (i.e., isoniazid, rifampin, pyrazinamide, or ethambutol). The prevalence of resistance to specific first-line drugs ranged from 16% for isoniazid to 3% for rifampin and 2% each for pyrazinamide and ethambutol. The prevalence of multidrug-resistant tuberculosis (MDRTB) (i.e., resistant to at least isoniazid and rifampin) was 3%. Resistance to rifampin occurred exclusively with MDRTB (Table).

**Table:** Characteristics of 239 Somali patients with extrapulmonary tuberculosis, Minnesota, 1993–2003*

Characteristic	No. (%)
Age, y	
<15	20 (8)
15–24	102 (43)
25–44	99 (41)
45–64	10 (4)
>65	8 (3)
Sex	
Male	111(46)
Female	128 (54)
Immigration status	
Refugee	178 (74)
Other immigrant	16 (7)
Other/unknown	45 (19)
TST status (n = 210)	
Positive	201 (96)
Negative	9 (4)
HIV status (n = 179)	
Positive	2 (1)
Negative	177 (99)
Culture status, *Mycobacterium tuberculosis* (n = 239)	
Positive	197 (82)
Negative	42 (18)
Drug resistance (n = 197)	
Any first-line drug†	35 (18)
INH	32 (16)
RIF	5 (3)
EMB	4 (2)
PZA	4(2)
MDRTB‡	5 (3)

*TST, tuberculin skin test; INH, isoniazid; RIF, rifampin; EMB, ethambutol; PZA, pyrazinamide; MDRTB, multidrug-resistant tuberculosis. †INH, RIF, EMB, PZA. ‡Resistant to at least INH and RIF.

Of the 186 patients who had extrapulmonary tuberculosis and were treated from 1993 through 2002, 91% successfully completed an adequate course of therapy, 76% within 12 months. Of the 186 treated patients, 55% received strict directly observed therapy, and 25% others received a less intensive form of supervised therapy. Response to treatment did not differ significantly between patients who received directly observed therapy or some other form of supervision and those who administered therapy themselves. One patient died, a 9-year-old girl with MDRTB in multiple sites.

## Conclusions

Extrapulmonary tuberculosis is more common than pulmonary tuberculosis in Somalis in Minnesota. Among Somali patients who have extrapulmonary tuberculosis, 50% have lymphatic disease, most are <45 years of age, and slightly more are female; prevalence of HIV infection is low, prevalence of reactive tuberculin skin tests is high, and prevalence of drug-resistant strains is substantial. These findings are similar to those reported for Somali immigrants in other countries (*10*). The prevalence of extrapulmonary tuberculosis among all foreign-born tuberculosis patients in the United States is considerably lower than that reported among Somalis in Minnesota and elsewhere (*11*), which suggests that the unique characteristics of tuberculosis in this population may reflect host factors or differences in geographically endemic strains of *M. tuberculosis*.

In Minnesota, Somali patients who had extrapulmonary tuberculosis were older and had resided in the United States longer than Somali patients who had pulmonary tuberculosis. These differences likely reflect the relative difficulty in diagnosing extrapulmonary disease compared with pulmonary tuberculosis. To minimize the interval between clinical manifestation of disease and diagnosis, clinicians should maintain an increased level of suspicion for extrapulmonary tuberculosis in Somali patients.

Immigration is a major factor in sustaining tuberculosis disease in the United States. This study demonstrates how immigration can affect the local epidemiology of tuberculosis through importation of disease patterns from another part of the world.
